# Development of SSR markers for genetic diversity analysis and species identification in *Polygonatum odoratum* (Mill.) Druce based on transcriptome sequences

**DOI:** 10.1371/journal.pone.0308316

**Published:** 2024-09-23

**Authors:** Gen Pan, Jing Xie, Yuhui Qin, Shuihan Zhang

**Affiliations:** 1 Institute of Chinese Medicine Resources, Hunan Academy of Chinese Medicine, Changsha, Hunan Province, China; 2 Colleges of Chinese Medicine, Hunan University of Chinese Medicine, Changsha, Hunan Province, China; 3 Institute of Bast Fiber Crops, Chinese Academy of Agricultural Sciences, Changsha, Hunan Province, China; Sant Baba Bhag Singh University, INDIA

## Abstract

*Polygonatum odoratum* (Mill.) Druce is a well-known traditional Chinese herb belonging to the *Polygonatum*. However, the understanding of the genetic diversity of this species at the molecular level is limited due to the lack of transcriptomic and genomic information. In this study, 37,387 unigenes were assembled based on the transcriptome sequencing of the rhizome of *Polygonatum odoratum* (Mill.) Druce., and 11,021 single- sequence repeats (SSR) motifs, mainly consisting of single-nucleotide repeats (44.44%), dinucleotides (31.06%), and trinucleotides (22.59%), were identified. Based on these SSR motifs, 9,987 primer pairs of SSR markers were designed and 68 SSR markers were randomly selected for verification, of which 21 SSR markers showed polymorphisms among the 24 *Polygonatum odoratum* germplasms. Ninety-four alleles were detected: the observed alleles ranged from 2 to 11, the effective alleles varied from 1.086 8 to 4.916 8, the Shannon diversity index was 0.173 2~1.749 7, and the polymorphism information content PIC ranged from 0.076 7 to 0.803 9. Based on our analysis of genetic diversity (SSR genotypes) and population structure, we divided the 24 germplasm resources into two groups, indicating that the germplasm with similar geographical origins can be grouped together. In addition, the primers ‘YZ14’ and ‘YZ47’ could effectively distinguished the related species: *Polygonatum kingianum* Coll.et Hemsl., *Polygonatum sibiricum* Red., *Polygonatum cyrtonema* Hua, *Polygonatum zanlanscianense* Pamp. and *Polygonatum odoratum* (Mill.) Druce. This is the first study in which a dataset of expressed sequence tag (EST)-SSR markers is constructed for the *Polygonatum odoratum* (Mill.) Druce, and these newly developed EST-SSR markers provided a very efficient tool for genetic relationship analysis, species identification and marker-assisted selection breeding of *Polygonatum odoratum* (Mill.) Druce.

## Introduction

*Polygonatum odoratum* (Mill.) Druce (*P*. *odoratum*), a species in the monocot family Asparagaceae, is used as a traditional Chinese medicine and is widely distributed in Asia, including China and the other Southeast Asian countries such as Thailand and Vietnam [1]. In China, *P*. *odoratum* is an important resource for traditional Chinese medicine and the healthcare industry because of its pharmacological effects such as lowering blood sugar and blood lipids and regulating immunity [2]. In addition, as a plant used for both medicine and food, *P*. *odoratum* can be used as tonic tea and *P*. *odoratum* Congee, and *P*. *odoratum* Snow are used in mushroom soup. China has abundant wild and cultivated germplasm resources of *P*. *odoratum*, which are widely distributed in Heilongjiang, Jilin, Hubei, Hunan, and other regions.

In recent years, owing to the continuing deepening of research on the medicinal and edible value of *P*. *odoratum*, its market demand has increased, resulting in a large amount of wild resources of *P*. *odoratum* plants being harvested and some germplasm resources being on the verge of extinction. In addition, owing to genetic variation and interspecific hybridization, it is difficult to discern species within the genus *Polygonatum* solely by phenotypic characteristics [3, 4]. Consequently, there are many counterfeit products on the market, and the quality of medicinal materials is extremely unstable, compromising drug safety. As an example, the leaves of *P*. *odoratum* and *Polygonatum cyrtonema* Hua (*P*. *cyrtonema*) are both mutualistic, making it difficult to distinguish between them at the seedling stage. Similarly, the rhizomes of *P*. *odoratum* may be misidentified as the rhizomes of *Polygonatum sibiricum* Red (*P*. *sibiricum*) because of their similar rhizome shapes, although they have different pharmacological properties. Therefore, it is necessary to assess the genetic diversity of *P*. *odoratum* and establish a molecular identification technology to distinguish easily confused Chinese medicinal materials in the genus *Polygonatum* and to provide a reference basis for the protection of germplasm resources, variety selection, and commodity identification of *Polygonatum*.

In plants, molecular markers can reveal the relationships between and within species at the DNA level, and can be used to discriminate between species regardless of their environment and morphology [5]. DNA markers have been widely used to analyse genetic diversity and identify species identification in medicinal plants, such as Cannabis, *Polygonatum Mill*., and *Physalis* species [3, 6, 7]. More than 20 types of DNA markers have been developed, and among which simple sequence repeats (SSRs) are recognized as the best choice for analyzing genetic diversity because of their high variability, codominance, reproducibility, and cross-species transferability [8]. SSRs have been widely used for genetic diversity analysis and species identification in crops, such as tea plants [9], Chinese yam [10], and palmae species [11]. They can be divided into expressed sequence tag SSRs (EST-SSRs) and genomic SSRs (g-SSRs). In general, EST-SSRs have been identified in the transcribed RNA sequences, and genomic SSRs (g-SSRs) have been developed based on their genomic sequences [12]. However, the genome of *P*. *odoratum* has not yet been sequenced, and transcriptome sequencing has become the only source of SSR molecular markers. To date, only a transcriptome of *P*. *odoratum* has been reported [13], which limits the development and application of SSRs in molecular research on *P*. *odoratum*.

In this study, we analysed and screened the distribution characteristics of SSR markers in the transcriptome sequence of rhizome of *P*. *odoratum*. SSR primers were designed and validated by evaluating the genetic diversity of 24 germplasms, and species identification in the genus *Polygonatum* were performed. Our study provides a useful tool for the genetic diversity analysis, species identification, and molecular marker-assisted breeding of *P*. *odoratum*.

## Materials and methods

### Plant materials and DNA extraction

The plant material was collected from five provinces in China; the cultivars were verified by Associate Professor Gen. Pan at the Institute of Chinese Medicine Resources, Hunan Academy of Chinese Medicine. The 28 *Polygonatum* germplasms, including 24 *Polygonatum odoratum* L., 1 *Polygonatum kingianum* Col et Hemsl., 1 *Polygonatum sibiricum* Red., 1 *Polygonatum cyrtonema* Hua, and 1 *Polygonatum zanlanscianense* Pamp., were planted at Wangcheng Base (Institute of Chinese Medicine Resources, Hunan Academy of Chinese Medicine). Detailed information is presented in Table 1. Rhizomes of the ‘A4’ germplasm were collected at the mature stage, then flash-frozen in liquid nitrogen and stored in a freezer at -80°C for subsequent RNA extraction and transcriptome sequencing. Three biological replicates of the rhizome tissue were prepared.

**Table 1:** The detailed information of germplasms resources in the *Polygonatum*.

Name	herbarium numbers	The Geographical Place of Origin (China)	Species	Types
A1	2021-C1	Xupu Country, Huaihua City, Hunan Province	*Polygonatum odoratum*	Cultivated
A2	2021-C2	Xupu Country, Huaihua City, Hunan Province	*Polygonatum odoratum*	Cultivated
A3	2021-C3	Guiyang Country, Chenzhou City, Hunan Province	*Polygonatum odoratum*	Cultivated
A4	2021-W1	Xinhua Country, Loudi City, Hunan Province	*Polygonatum odoratum*	Wild
A5	2021-C4	Ji’ang Country, Tonghua City, Jilin Province	*Polygonatum odoratum*	Cultivated
A6	2021-C5	Hengren Country, Benxi City, Liaoning Province	*Polygonatum odoratum*	Cultivated
A7	2021-W2	Hao Country, Luoyang City, Henan Province	*Polygonatum odoratum*	Wild
A8	2021-C6	Cili Country, Zhangjiajie City, Hunan Province	*Polygonatum odoratum*	Cultivated
A9	2021-C7	Anhua Country, Yiyang City, Hunan Province	*Polygonatum odoratum*	Cultivated
A10	2021-W3	Danjiangkou Country, Shiyan City, Hubei Province	*Polygonatum odoratum*	Wild
A11	2021-W4	Xunyang Country, Ankang City, Shaanxi Province	*Polygonatum odoratum*	Wild
A12	2021-W5	Dongkou Country, Shaoyang City, Hunan Province	*Polygonatum odoratum*	Wild
A13	2021-W6	Xinning Country, Shaoyang City, Hunan Province	*Polygonatum odoratum*	Wild
A14	2021-W7	Chengbu Country, Shaoyang City, Hunan Province	*Polygonatum odoratum*	Wild
A15	2021-W8	Wugang Country, Shaoyang City, Hunan Province	*Polygonatum odoratum*	Wild
A16	2021-W9	Guiyang Country, Chenzhou City, Hunan Province	*Polygonatum odoratum*	Wild
A17	2021-C8	Shaodong Country, Shaoyang City, Hunan Province	*Polygonatum odoratum*	Cultivated
A18	2021-C9	Xupu Country, Huaihua City, Hunan Province	*Polygonatum odoratum*	Cultivated
A19	2021-C10	Xupu Country, Huaihua City, Hunan Province	*Polygonatum odoratum*	Cultivated
A20	2021-C11	Xupu Country, Huaihua City, Hunan Province	*Polygonatum odoratum*	Cultivated
A21	2021-C12	Lianzhou Country, Qingyuan City, Guangdong Province	*Polygonatum odoratum*	Cultivated
A22	2021-C13	Xishui Country, Suizhou City, Hubei Province	*Polygonatum odoratum*	Cultivated
A23	2021-C14	Sangzhi Country, Zhangjiajie City, Hunan Province	*Polygonatum odoratum*	Cultivated
A24	2021-W10	Suining Country, Shaoyang City, Hunan Province	*Polygonatum odoratum*	Wild
A25	2021-W11	Maojian District, Shiyan City, Hubei Province	Polygonatum zanlanscianense Pamp.	Wild
A26	2021-W12	Nanyue District, Hengyang City, Hunan Province	*Polygonatum cyrtonema* Hua	Wild
A27	2021-C15	Acheng District, Harbin City, Heilongjiang Province	*Polygonatum sibiricum* Red	Cultivated
A28	2021-C16	Xupu Country, Huaihua City, Hunan Province	*Polygonatum kingianum*	Cultivated

DNA was extracted from the rhizomes of 28 materials using a Plant Genome DNA Extraction Kit (Tiangen Biochemical Technology, Beijing, China) according to the manufacturer’s instructions. The quality and concentration of the total genomic DNA were determined using a NanoDrop 2000 ultra-micro spectrophotometer (Thermo Fisher Scientific, Waltham, MA, USA). Qualified genomic DNA stock solutions were diluted to 50 ng/μL for analysis via polymerase chain reaction (PCR).

### Total RNA extraction, cDNA library construction, sequencing and analyses

Total RNA was extracted from the rhizomes of ‘A4’ *P*. *odoratum* using an EASYspin Plus Plant RNA Kit (Aidlab Biotechnologies Co., Ltd., Beijing, China). The quality and quantity of the total RNA was assessed on agarose gels and determined using a NanoDrop 2000 instrument (Thermo Fisher Scientific). One μg of total RNA from the rhizome of the ‘A4’ germplasm was then prepared for cDNA synthesis using a NEBNext® Ultra™ II RNA Library Prep Kit (New England Biolabs Inc., Ipswich, MA, USA) according to the previously published protocol [14]. A Qubit 2.0 fluorometer (Life Technologies, CA, USA) and a Bioanalyzer 2100 system (Agilent Technologies, Santa Clara, CA, USA) were used to verify the amount and purity of the library. Lastly, the RNA was sequenced using an Illumina NovaSeq 6000 platform (Illumina) supplied by Shanghai OE Biotech Co., Ltd. (Shanghai, China).

The raw data were processed and filtered using Trimmomat to remove reads containing multiple N’s and poor quality reads, resulting in clean data. The fragments per kilobase of the exon model per million mapped fragments (FPKM) value for each unigene were calculated using Cufflinks. To analyze the functionality of the unigenes, the NR, KOG, GO, Swiss-Prot, eggNOG, and KEGG databases were aligned using DIAMOND [15], and the Pfam databases were aligned using HMMER [16].

### Development and primer design of SSR markers

MISA (MIcroSatellite identification tool) software (http://pgrc.ipk-gatersleben.de/misa/) was used to identify microsatellite motifs and to cluster the compounding of microsatellite motifs with dimer, trimer, tetramer, pentamer and tetramer motifs (≥12 bp) in the *P*. *odoratum* transcriptome. Sequences with SSR motif flanking lengths ≥50 bp were selected for primer design using Primer 3.0 software (http://pgrc.ipk-gatersleben.de/misa/primer3.html).

### SSR genotyping

The 68 primer pairs were randomly selected from the 9,987 primers used for amplification validation. PCR analysis was performed using: 7 μL PCR mixed solution (Qingke, Nanjing, China), 1 μL forward primer (10 nmol/L), 1 μL reverse primer (10 nmol/L), and 1 μL DNA template. The PCR was conducted as follows: 95°C for 5 min, followed by 32 cycles of 45 s at 94°C, 30 s at the primer-specific annealing temperature, 60 s at 72°C, and a final extension of 5 min at 72°C. PCR products were detected by 8.0% non-denaturing polyacrylamide gel electrophoresis (PAGE). After running at 200V for 1.5 h, the PAGE gel was stained with a 0.1% AgNO_3_ solution.

### Data analysis

To construct a matrix of binary values (0 and 1), the polymorphism bands present using the different primers in a sample were recorded as ‘1’, while those absent were recorded as ‘0’. SSR marker characteristics, including observed alleles, effective alleles, and Shannon diversity index, were analysed using the software PopGen 1.32, and polymorphism information content was calculated using PowerMarker 3.25. A clustering map was constructed using NTSYSpc 2.11 based on genetic distances and the unweighted pair group method with arithmetic mean (UPGMA), and the simple matching (SM) coefficient was used to construct a tree. STRUCTURE v2.3.4 was used to estimate the population structure of the 24 genotypes of the *P*. *odoratum* germplasm, and the number of subpopulations (K) was set from 1 to 10 based on admixture models and run 20 times per round.

## Results

### *De novo* transcriptome assembly

**Fig 1:**
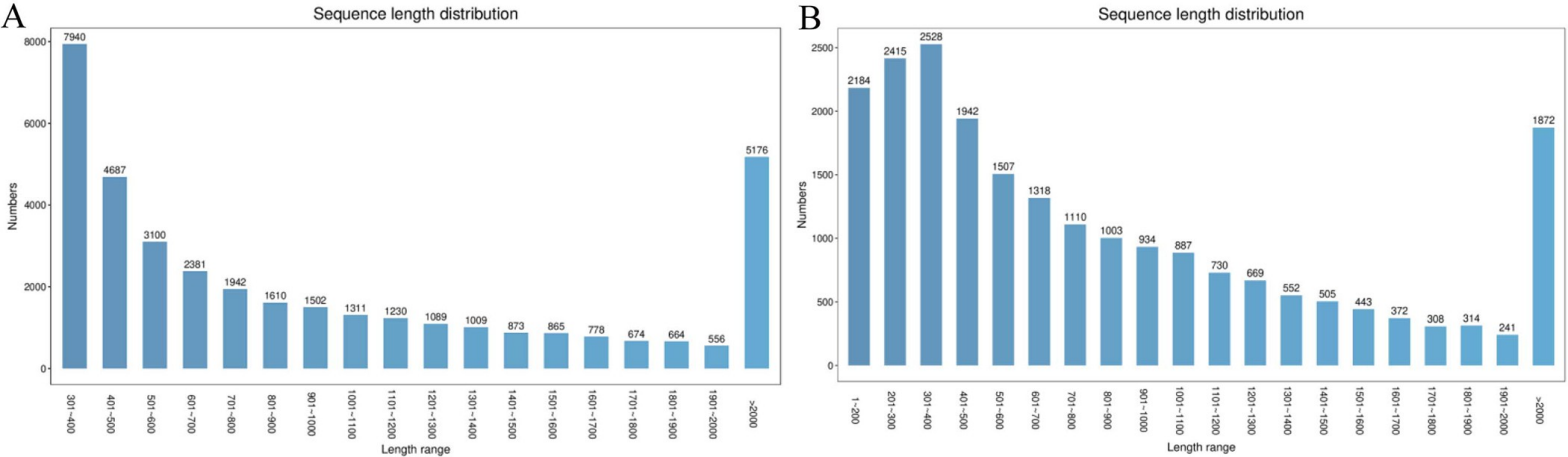
The number of unigenes and CDS sequence length of *Polygonatum odoratum* transcriptome distributed in different length range. **(A)** The number of unigenes sequence length distribution. **(B)** The number of CDS sequence length distribution.

Transcriptome sequencing of the rhizome of ‘A4’ yielded 21.71 of clean data, and the amount of clean data for three replicates ‘A4-1’, ‘A4-2’ and ‘A4-3’ were 7.08 G, 7.5 G, and 7.13 G, respectively, with a Q30 base distribution of 90.6%~93.67%, and a GC content of 47.29%~48.24% (Table 2). In total, 37,387 unigenes were obtained with a total length of 40,524,982 bp. The average length of the 37,387 unigenes was 1083.93 bp, and the largest number of unigenes length was 301–400 bp range, followed by >2,000 bp range (Fig 1A). Among these unigenes, the maximum length was 13,638 bp and the minimum length was 301 bp, respectively (Table 3). In addition, 26,873 CDS sequences were predicted based on 37,387 unigenes, and the largest number of CDS sequences was 301–400 bp (2528), followed by 301–400 bp and 1–200 bp (Fig 1B).

**Table 2:** Information of transcriptome of ‘A4’ of *Polygonatum odoratum*.

Sample	RawReads(M)	RawBases(G)	CleanReads(M)	CleanBases(G)	ValidBases(%)	Q30(%)	GC(%)
A4-1	48.07	7.21	47.99	7.08	98.17	93.51	47.9
A4-2	50.73	7.61	50.66	7.5	98.52	93.67	47.29
A4-3	48.28	7.24	48.21	7.13	98.4	90.6	48.24

**Table 3:** RNA-sequencing data of *Polygonatum odoratum*.

Description	Statistics
Unigene	37,387
> = 500bp	24,790
> = 1000bp	14,237
N50	1585
Total_Length	40,524,982
Max_Length	13,638
Min_Length	301
Average_Length	1083.93

### Unigene annotation

**Fig 2:**
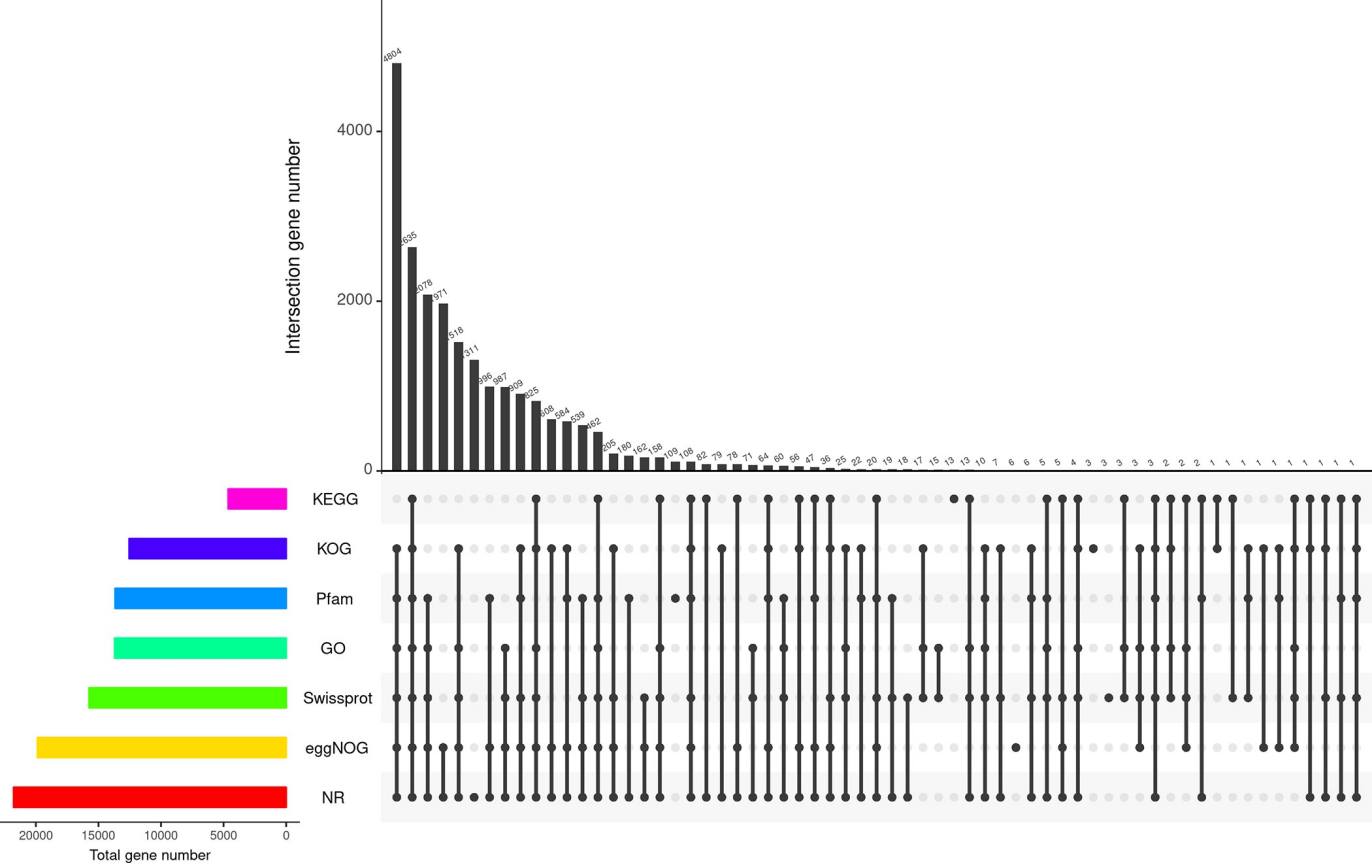
A Venn diagram of 7 annotation database. The numbers on the top bar represent the results of the intersection of the databases in the matrix below corresponding to the databases with black dots, and the bars on the left represent the number of unigenes fully annotated to each database.

Functional analysis of the unigene was performed by comparing them to the NR, KOG, GO, Swiss-Prot, eggNOG, and KEGG databases using the Diamond and to Pfam databases using HMMER. In total, 21,776 unigenes (58.24%) were annotated in the NR database, 15,742 (42.11%) in the Swissprot database, 4630 (12.38%) in the KEGG database, 12,541 (33.54%) in the KOG database, 19,869 (53.14%) in the eggNOG database, 13,701 (36.65%) in the GO database, and 13,666 (36.55%) in the Pfam database (Fig 2). Furthermore, 4,804 unigenes were annotated in all seven databases (Fig 2).

### Identification and characterization of SSRs

**Fig 3:**
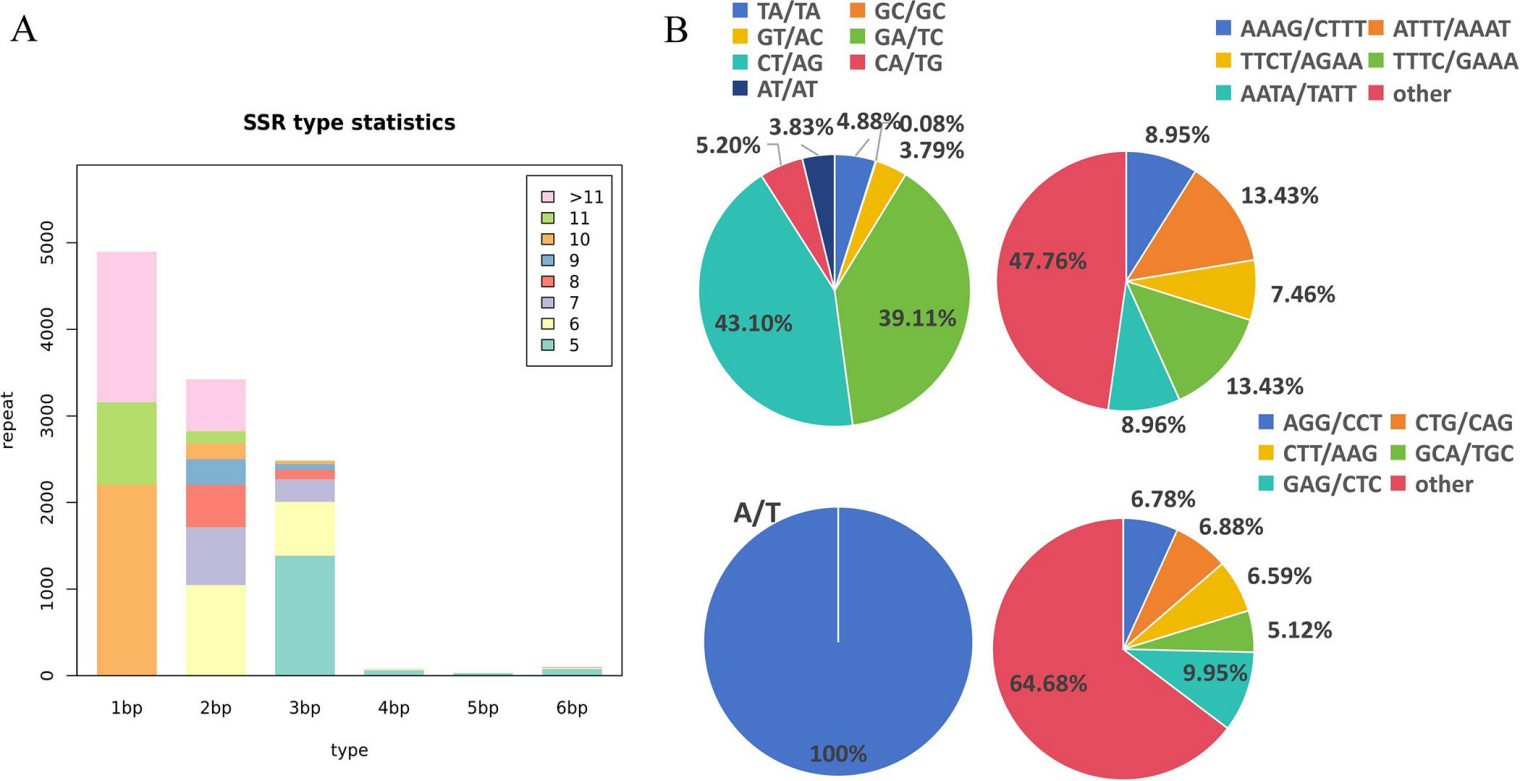
Characteristics of SSRs motif of *Polygonatum odoratum* transcriptome. **(A**) SSR type statistics chart. **(B)** Proportion distribution of different type SSR motifs.

Based on the transcriptome of ‘A4’ rhizomes, a total of 11,021 SSR motifs, including 1,034 compound SSRs, were detected in 8,311 unigenes using MISA. Among these SSR motifs, single-nucleotide repeats were the most common, accounting for 44.44% of the total SSRs (4,898), followed by 3,423 dinucleotide and 2,490 trinucleotide SSRs, accounting for 31.06% and 22.59%, respectively, of the total SSRs. Pentanucleotides (33) constituted the smallest proportion of all types of SSR motifs, accounting for only 0.3% of the total SSRs (Fig 3A). All the single-nucleotide repeat SSRs were A/T. Among the dinucleotide repeats, the percentage of CT/AG was the highest, whereas that of GC/GC was the lowest, comprising 27.83% and 0.02%, respectively, of the total (Fig 3B). Among the trinucleotide repeats, five types of SSRs accounted for more than 5%: AGG/CCT, CTG/CAG, CTT/AAG, GCA/TGC and GAG/CTC. With regard to the tetranucleotides, the two types of SSRs (ATTT/AAAT, and TTTC/GAAA) had the highest frequencies (Fig 3B). Additionally, pentanucleotide and hexanucleotide repeats were more difficult to distinguish because they represented only a small proportion of the total SSRs detected.

### Transcriptome-wide SSR marker development and amplification validation

**Fig 4:**
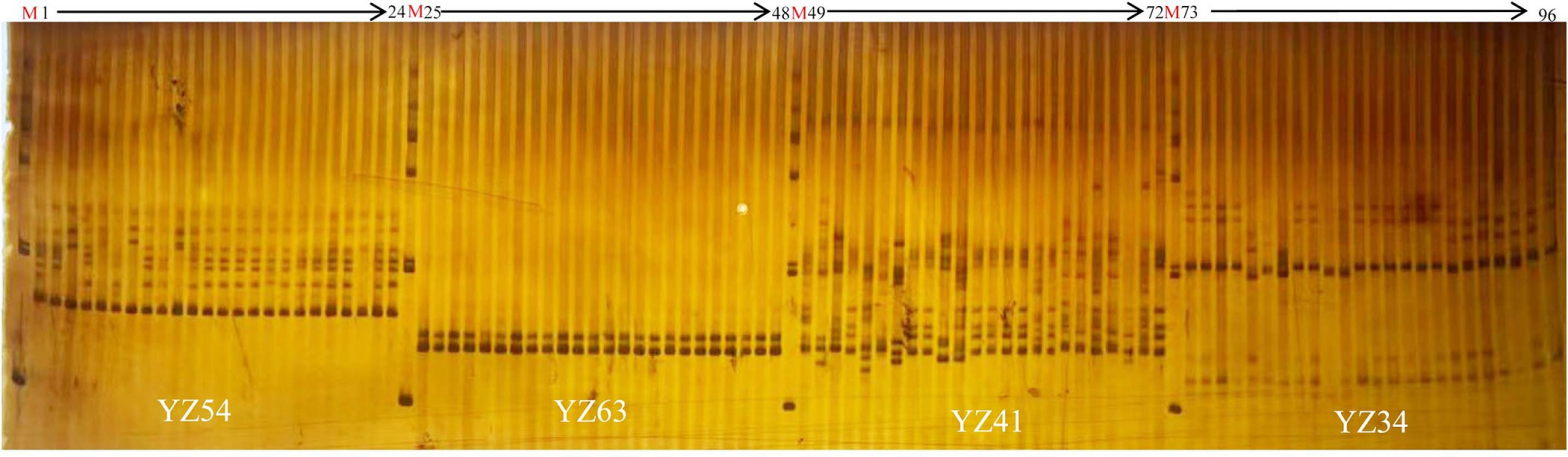
Amplification results from 24 *Polygonatum odoratum* germplasms using the SSR markers *‘*YZ41’, ‘YZ54’ and ‘YZ63’. The SSR markers ‘YZ54’ and ‘YZ41’ showed polymorphism among 24 *Polygonatum odoratum* germplasm resources.

A total of 9,987 primer pairs with a length of 20 bp were successfully designed based on 11,021 SSR motifs, and their target product size ranged from 100 to 300 bp. Subsequently, 68 pairs of SSR primers were randomly selected for validation. The results showed that 41 SSR markers effectively amplified the target bands (S1, S2 Figs), and 21 pairs of primers were found to be polymorphic among the 24 germplasms. The number of amplified fragments ranged from 2 to 11. The primer with the highest number of amplified bands was ‘YZ41’ (11) (Fig 4), and the primers with the lowest number of amplified bands were ‘YZ32’ and ‘YZ56’.

### Genetic diversity analysis of *P*. *odoratum* germplasms

The 41 SSR markers that effectively amplified the target bands were used to analyse the genetic relationships. Twenty-one SSR markers amplified 94 polymorphic fragments, with an average of 4.47 polymorphic bands. In addition, the *PIC* ranged from 0.076 7 (‘YZ32’) to 0.803 9 (‘YZ14’), with an average of 0.432 7. The number of observed alleles *Na* ranged from 2 to 11, and the marker with the highest number of observed alleles was ‘YZ41’, with an average number of observed alleles of 4.476 1. The number of effective alleles *Ne* ranged from 1.086 8 to 4.916 8, with the highest number of marker being ‘YZ14’, and the lowest number of marker being ‘YZ32’, with a mean of 2.337 8. The Shannon diversity index ranged from 0.173 2 (‘YZ32’) to 1.749 7 (‘YZ41’), with a mean value of 0.868 4 (Table 4).

**Table 4:** Genetic diversity analysis of *Polygonatum odoratum* germplasms using 21 pairs EST-SSR markers.

Marker ID	Repeat Motif	Forward Primer (5’-3’)	Reverse Primer (5’-3’)	*Na*	*Ne*	*I*	PIC
YZ8	(GA)6	CACGACAAGAAGCAGGTCCT	GGGACTGACACGTGTAAGCA	3	2.5974	1.0104	0.5339
YZ10	(GAA)5	GCACCGCCTCCTAAAGAAGT	TGGCCACTTGTAATGCGAGT	3	1.4382	0.4826	0.2583
YZ11	(CAG)6	ATTCACCAGTGCTCCGTCTG	AGTAGCGGTCAAAGTTCCGG	3	1.9459	0.6792	0.368
YZ14	(GTGCTG)7	AGCAGCAGCAGTAGTCACAG	TCAAGACTTTCCCGTCTGGC	8	4.9168	1.6713	0.8039
YZ15	(TGGAGC)5	TCGAGGGGCTGATGAAGGTA	AAAAATCAACACCCCGTGCG	3	1.2082	0.3145	0.1575
YZ20	(CAT)7	CTTAAACGAGGACCTCCGGC	GCCTATACCTCACCCCAAGC	6	3.0104	1.3169	0.611
YZ23	(TCC)5	CTCCATCCTCTCCAGTCGGA	GGACGGCATATGAAGGGGTT	4	2.3349	0.6176	0.5194
YZ25	(GAA)5	CTCTGCTGCTTCGTCCTGAA	GTCGCTATAGCCGAGGTCAG	5	2.0105	0.7619	0.3955
YZ28	(CT)9	GCGTACGATTCCTTTGGGGA	CAGCCGGGTCAATCATCGAT	5	1.9327	0.6338	0.3103
YZ32	(ATTTG)5	GAACGGCCTGTACACCTTCA	TGCTCCTCCCGTTCTTCAAC	2	1.0868	0.1732	0.0767
YZ33	(CAGTAG)5	CTTACCGCTCGACCACAAGT	GCAACTCGACTGCAACTTGG	3	1.5008	0.578	0.2912
YZ34	(AGG)5	TCCATCCCTGAGCTCCAGAA	AGTGTGGTGTTGCTTGTCGA	6	4.1654	1.5913	0.7259
YZ39	(TC)10	CATGGCCGAGCACAAAGAAG	CCTTGAAGAGGTGGCAGAGG	3	1.7664	0.775	0.3939
YZ41	(GAG)6	GTGCTCGTGATTCGGATTGC	CTCATTGTACCGCAGCAACG	11	4.7215	1.7497	0.7826
YZ47	(CCT)7	AACCAGCCTCCTCCTCATCT	TGCTGGGTAGGGGATGATCA	7	3.1138	1.5977	0.6791
YZ54	(AAG)5	CGTAGCCATCTCCCTTGCAA	TTTCTCCACGTACGTCAGGC	5	2.9887	1.2602	0.6084
YZ55	(AAG)5	TCAATTCTCCGCCTCCGATG	AGCGAGCGAAAACCTCTTCA	4	1.41	0.5608	0.2702
YZ56	(AAG)5	GGATTCGCAGTCCTCGAGAG	GCTTCGGGTTCCAAAACACC	2	1.9692	0.6853	0.3711
YZ62	(AAG)5	CATTGGCAGAATCAACGGGC	CATGAACTGCCCTCCCATGT	5	2.2088	0.8766	0.453
YZ68	(AAG)6	AGGCTGAAGAGAGCTCTGGA	GAGCATACGAGGAAGTCCGG	3	1.3846	0.4506	0.2392
YZ66	(AAG)6	ACCCCTACCAGTCCATCTCC	GAGGAGGAGGAGGAGACGTT	3	1.3846	0.4506	0.2392
Mean				4.4761	2.3378	0.8684	0.4327

*NA*: number of allele, *Ne*: number of effective allele, *I*: Shannon diversity index, *PIC*: polymorphic information content

**Fig 5:**
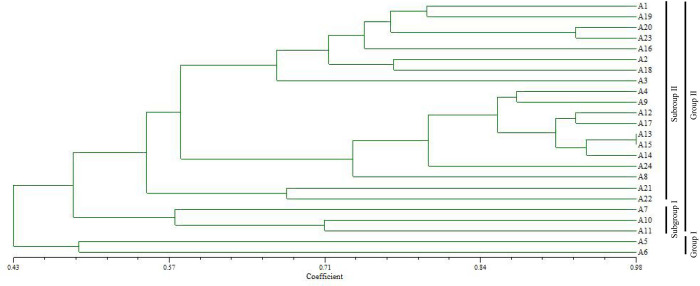
Phylogenetic tree of 24 *Polygonatum odoratum* germplasms using 21 SSR markers.

**Fig 6:**
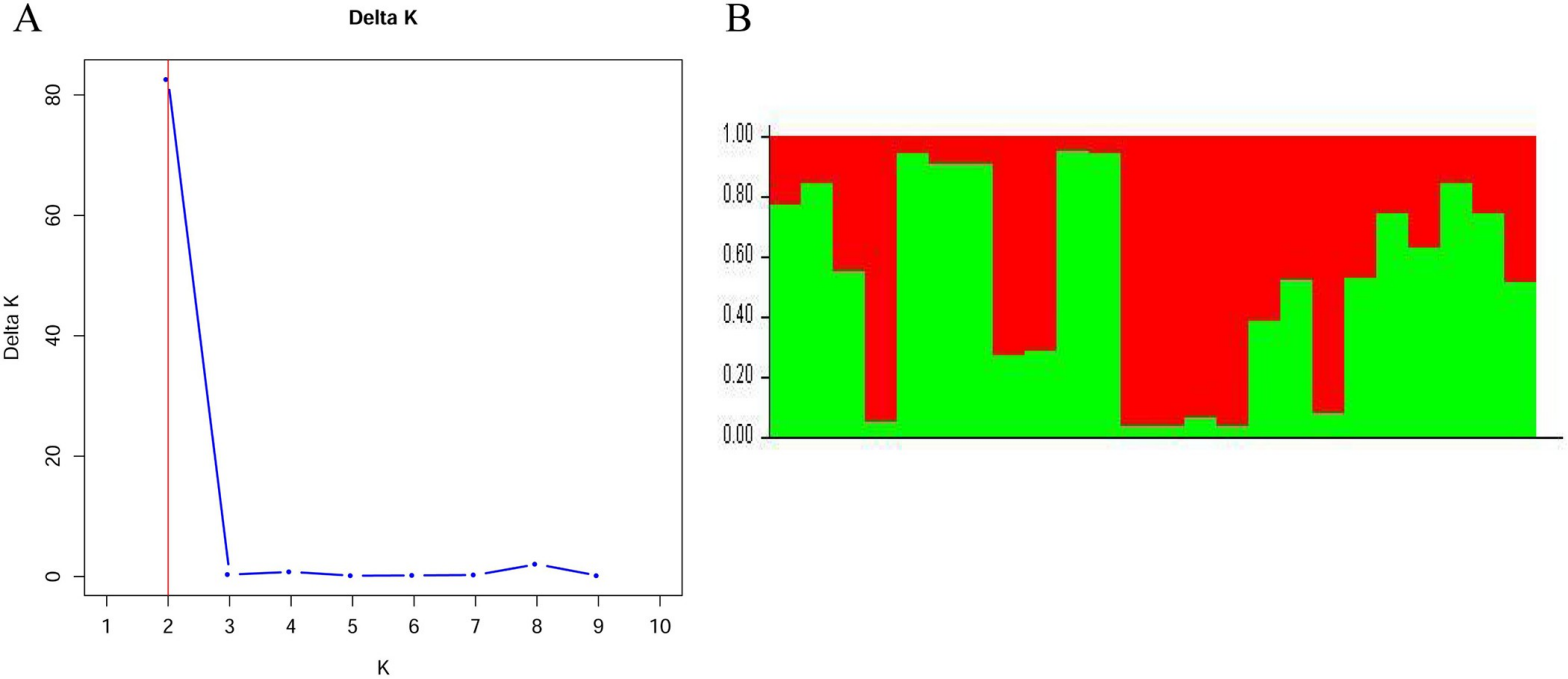
STRUCTURE analysis of the number of population for K. **(A)** The number of subpopulations (K) was identified based on maximum likelihoodand K values. The most likely value of k identified by STRUCTURE was observed at K = 2. **(B)** The proportion of each color reflects the probability that each of the 24 *Polygonatum odoratum* germplasms belongs the corresponding group based on 21 SSR markers (K = 2). Different colors represent different groups.

Based on the genotyping results obtained using 21 SSR markers, 24 *P*. *odoratum* germplasms were further analysed by clustering using the UPGMA method. As shown in Fig 5, the genetic similarity coefficients of 24 *P*. *odoratum* germplasms ranged from 0.43 to 0.98, with an average value of 0.705. At a genetic coefficient of 0.43, the 24 germplasms were divided into two major groups, in which the *P*. *odoratum* germplasms from the northeast region, ‘A5’ and ‘A6’, were clustered into one major group (Group Ⅰ), and the remaining 19 germplasms were clustered into another major group (Group Ⅱ). It was also found that the germplasms from the geographically neighbouring Nanyang in Henan Province, Shiyan in Hubei Province and Ankang in Shanxi Province were clustered into the same subgroup (Subgroup Ⅰ), whereas all the different germplasms, including the wild and cultivated germplasms from Hunan Province, were clustered into another subgroup (Subgroup Ⅱ) (Fig 5). As shown in Fig 6, Delta K reached a maximum value at K = 2, indicating that the 24 cultivars could be partitioned into two populations (Fig 6).

### Screening of SSR markers for species identification in the *Polygonatum*

**Fig 7:**
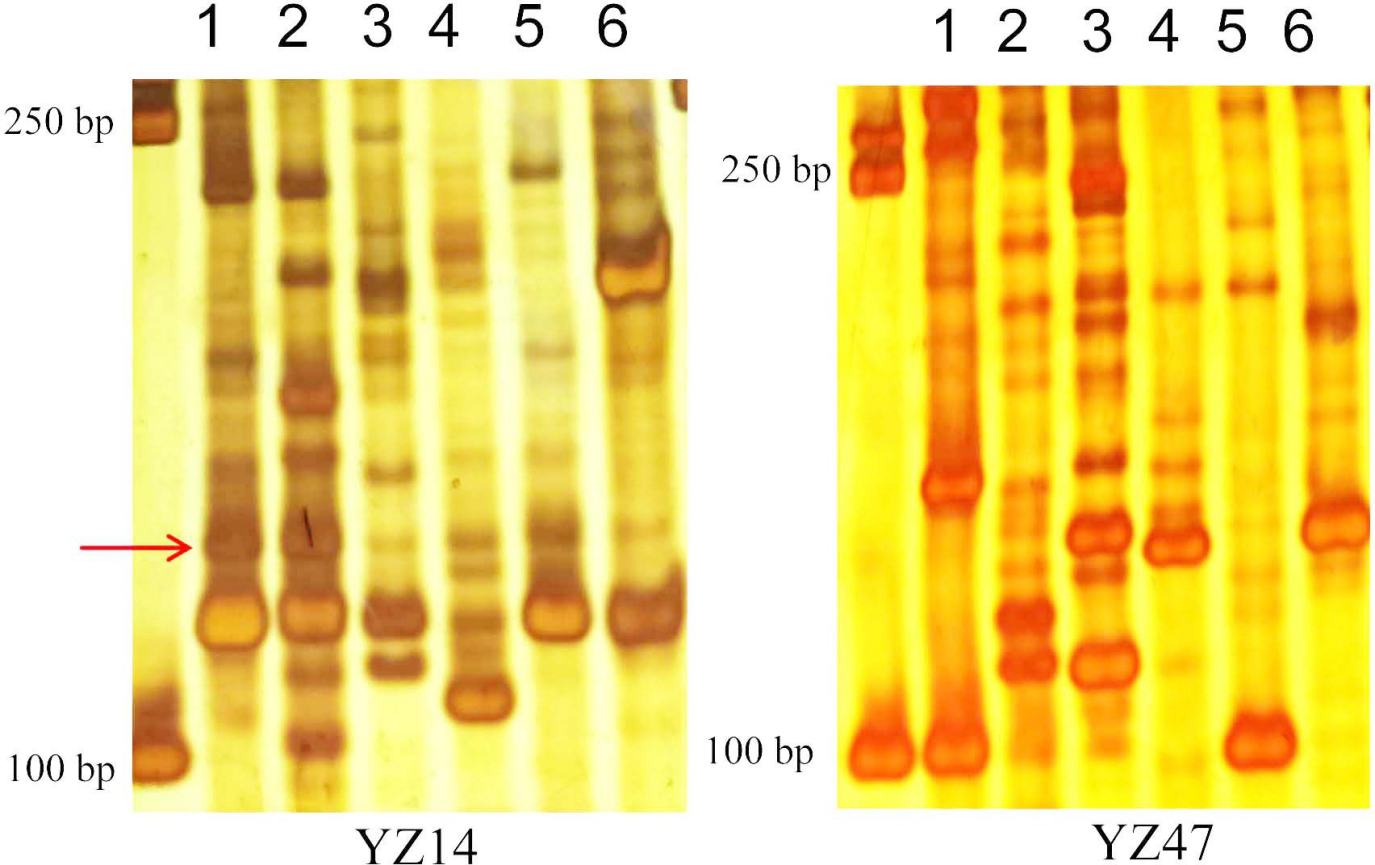
Image of polyacrylamide gel electrophoretic detection results of amplified products of five kinds in *Polygonatum* with ‘YZ14’ and ‘YZ47’ primers. M:DNA marker DL2000; 1: *Polygonatum odoratum* (Mill.) Druce (A17); 2: *Polygonatum odoratum* (Mill.) Druce (A5); 3:*Polygonatum zanlanscianense* Pamp. (A25); 4:*Polygonatum sibiricum* Red. (A27); 5: *Polygonatum sibiricum* hua (A26); 6: *Polygonatum kingianum* Coll.et Hemsl (A28).

The *Polygonatum* contains a variety of medicinal plants such as *P*. *kingianum* Coll.et Hemsl., *P*. *sibiricum* Red., *P*. *cyrtonema Hua*, *P*. *zanlanscianense* Pamp. and *P*. *odoratum*. Because the leaves of both *P*. *cyrtonema Hua* and *P*. *odoratum* are alternate, the appearance and morphology of the seedlings are similar, and it is more difficult to differentiate them from each other. Therefore, to provide the potential tool for species identification in the *Polygonatum*, 41 pairs of primers were used to screen specific molecular markers on the DNA of *P*. *kingianum* Coll.et Hemsl., *P*. *sibiricum* Red., *P*. *cyrtonema Hua*, *P*. *zanlanscianense* Pamp. and *P*. *odoratum*, respectively. As shown in Fig 7, two SSR markers (‘YZ14’ and ‘YZ47’) were obtained, which could specifically and effectively differentiate the above five types of Chinese herbs in the *Polygonatum*. It was also found that there was a specific amplified fragment in the ‘YZ14’ (indicated by the arrow in Fig 7), which distinguishes *P*. *odoratum* from the other four species (Fig 7).

## Discussion

As transcriptome sequencing technology continues to be developed, the cost of this technology has been lowered, making it possible to obtain the sequence information of species transcripts in a more comprehensive way; for example, in the development of EST-SSR molecular markers in a variety of medicinal plants [7,10, 17–19]. The detection of EST-SSRs depends on many factors including genomic structure, tools, and methods [20]. In this study, 11,021 EST-SSR loci were identified in the transcriptome sequence of *P*. *odoratum* rhizome tissues with a distribution frequency of 29.47% per unigene (Fig 3), which was lower than that of *Amomum tsaoko* (37.83%) [19] and Chinese yam (36.44%) [10], but higher than that of cannabis (14.16%) [7], ginger (21.25%) [17] and Orchidaceae (11.18%) [21]. In contrast to other plants, an extremely high proportion of single-nucleotide SSRs (44.4%) was obtained in this present study. Excluding single-nucleotide repeat types, dinucleotides accounted for the largest proportion of the total polynucleotides (55.90%), which was similar to the sequencing results of sesame [22] and sweet potato [23], but different from those of Chinese yam [10] and Orchidaceae [21].

As the genome of *P*. *odoratum* has not yet been sequenced and there is only one report on transcriptome sequencing [13], our understanding of the genetic basis of this species is still largely unknown. To the best of our knowledge, this study is the first large-scale development of molecular markers in *P*. *odoratum*. Our results show that the primer amplification efficiency of the primers selected randomly was 60.2% in *P*. *odoratum*, which was higher than that in *Ligusticum* chuanxiong [24], but lower than that in *Lycium barbarum* [25]. In addition, the average *Na* and *PIC* values of the test primers in this study were 4.4761 and 0.4327, respectively (Table 4), which were higher than those reported for *Polygonatum cyrtonema Hua* [26]. These results indicated that the EST-SSR markers screened in this study were highly polymorphic and could be further used for authenticity identification and genetic diversity analysis.

Genetic distance can directly reveal the genetic diversity of different germplasms and indirectly reflect the similarities in their genetic backgrounds. In previous studies, researchers have mainly focused on the genetic diversity of *P*. *odoratum* germplasms in some regions, such as Hunan Province [27], Anhui Province [28] and Dalian City, Liaoning Province [29]. In the present study, the germplasm resources were obtained from 19 counties in seven provinces in China. Thus, the results are more representative of the diversity of *P*. *odoratu* (Table 1). Through UPGMA clustering based on genetic distances, we classified the *P*. *odoratum* germplasms into two clusters, and those with similar geographical origins can be better grouped together (Fig 5). Similar to the findings in a previous study on *P*. *odoratum* [30], these results showed that the clustering analysis based on EST-SSRs showed a certain degree of regionality, and some germplasms also showed the phenomenon of ‘large heterogeneity’, which may be related to the introduction of germplasms from different regions. In addition, the wild and cultivated germplasms from Hunan Province were clustered into subgroup Ⅱ (Fig 5), indicating that these germplasm resources are closely related and that the cultivars may have been domesticated from wild resources. In the future, we will increase the collection of germplasm resources in order to more fully understand the genetic relationship of *P*. *odoratum* germplasms in China. As well, there is a need to systematically evaluate the genetic diversity of the germplasm resources by combining phenotypic traits and molecular markers, in order to provide a basis for the genetic improvement and utilization of the *P*. *odoratum* germplasms.

Owing to the transitional morphology and overlapping geographical distribution of the *Polygonatum*, as well as the similar morphology of the medicinal parts, there is confusion regarding the medicinal use of the same genus of Chinese medicinal herbs after processing [31]. In this study, two SSR markers were obtained that specifically distinguished five different Chinese medicinal materials in the *Polygonatum*: *P*. *kingianum* Coll.et Hemsl., *P*. *sibiricum* Red., *P*. *cyrtonema Hua*, *P*. *zanlanscianense* Pamp. and *P*. *odoratum* (Fig 7). These molecular markers can be used as an effective complement to the morphological, microscopic observation, and physicochemical methods to identify the *Polygonatum*, especially in terms of the quality control of dispensing granules. Because of the limited number of plant materials used for molecular identification in this study, in particular that there was only one germplasm each of *P*. *kingianum* Coll.et Hemsl., *P*. *sibiricum* Red., *P*. *cyrtonema Hua*, and *P*. *zanlanscianense* Pamp., future research will focus on the validation of the newly developed ESR-SSR markers to meet the increasing identification needs of germplasm resource collection. The number of test germplasms of the five different species of *Polygonatum* will be increased in order to verify the universality of the above specific primers in the identification of traditional Chinese medicine. Interestingly, primer ‘YZ14’ was designed from the transcript sequence of the UDP-glucuronic acid transferase gene, which encodes a glycosyltransferase that is involved in polysaccharide biosynthesis [32]. Based on the results of this study, we speculate that this gene may be a marker gene to distinguish the five different Chinese medicinal herbs in the *Polygonatum*, which also needs to be further investigated in the future.

## Conclusion

In this study, 37,387 unigenes were obtained by sequencing the rhizome transcriptome of *P*. *odoratum*, from which 11,021 EST-SSR loci mainly consisting of single-nucleotide repeats, dinucleotides and trinucleotides were identified, and their characteristics were further analysed. In total, 9,987 primer pairs were successfully designed based on these loci, and 24 germplasm resources were clustered into two groups with a certain geographical pattern using 21 polymorphic SSR markers. Simultaneously, two pairs of molecular markers were obtained that could specifically identify the five types of Chinese herbal medicinal materials in *Polygonatum*. The results of this study not only provide a valuable reference for the genetic analysis of the *P*. *odoratum* germplasm resources, a useful tool to distinguish the confusing Chinese herbal medicines in the *Polygonatum*, but also enrich the number of molecular markers in *P*. *odoratum*, which can serve as a resource for molecular-assisted breeding and QTL mapping.

## Supporting information

S1 Fig Phenotypes of the growth and rhizome of *Polygonatum odoratum*. (TIF)

S2 Fig Amplification validation of EST-SSR primers. Different lanes represent different PCR products using different EST-SSR primers.
(TIF)

S1 Dataset (XLS)
