# Supplementary figures and images for: Development of SSR markers for genetic diversity analysis and species identification in Polygonatum odoratum (Mill.) Druce based on transcriptome sequences
Source: PLoS One. 2024 Sep 23;19(9):e0308316. doi: 10.1371/journal.pone.0308316 (PMC11419394; doi:10.1371/journal.pone.0308316)

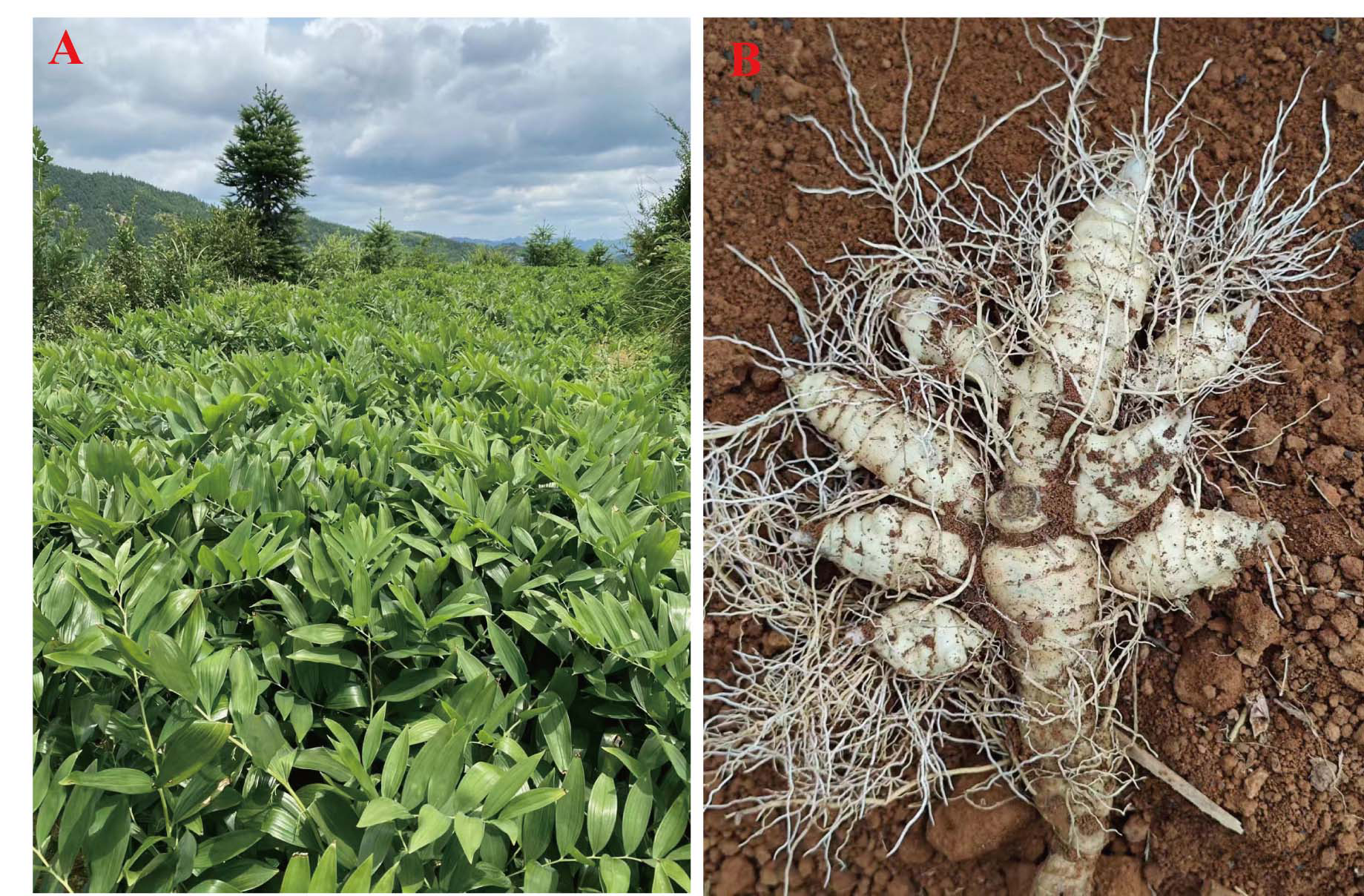

Supplement: S1 Fig — (TIF) [file pone.0308316.s001.tif]

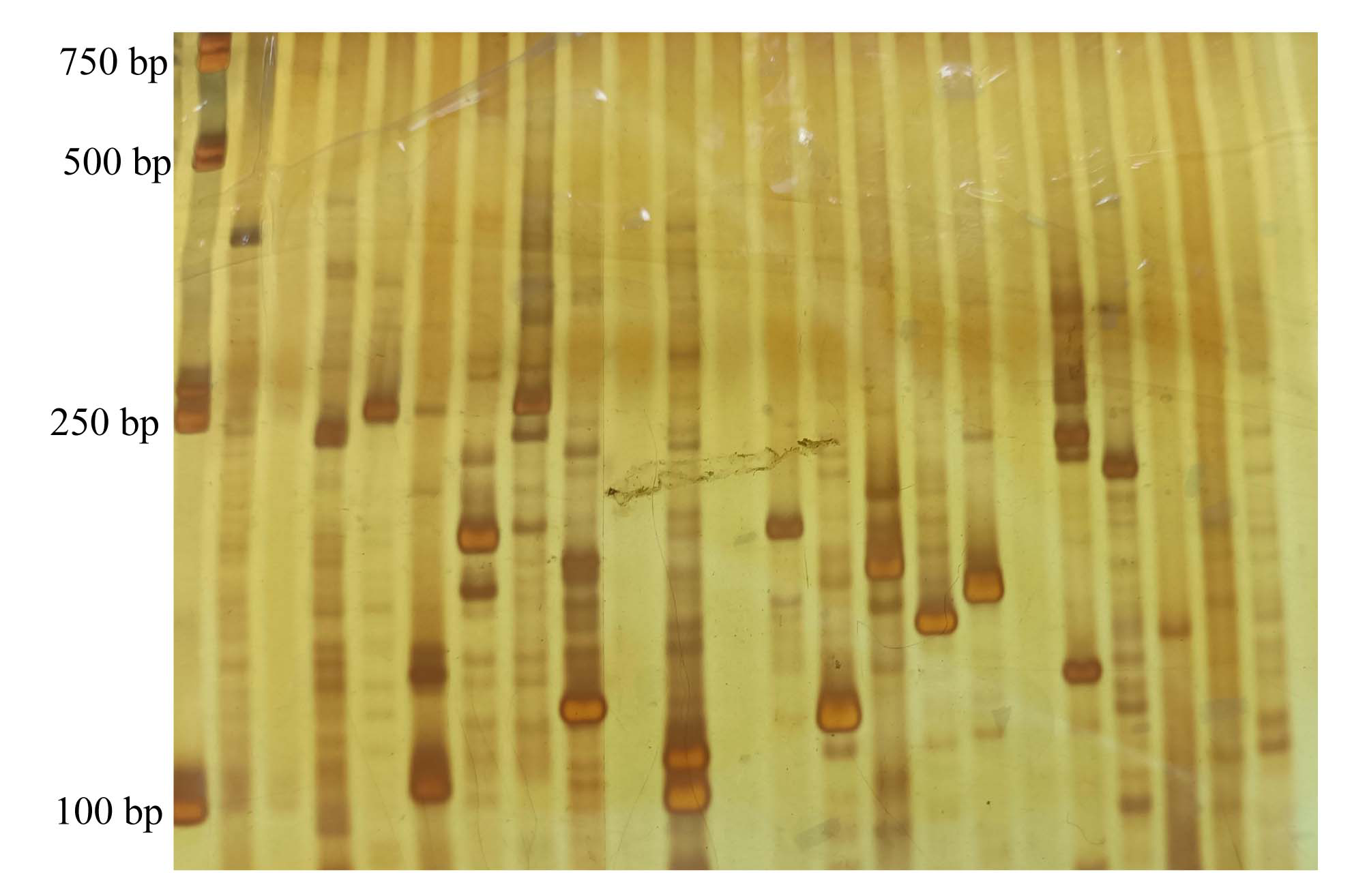

Supplement: S2 Fig — Different lanes represent different PCR products using different EST-SSR primers. (TIF) [file pone.0308316.s002.tif]
